# Facilitating Adaptive Emotion Processing and Somatic Reappraisal *via* Sustained Mindful Interoceptive Attention

**DOI:** 10.3389/fpsyg.2021.578827

**Published:** 2021-09-08

**Authors:** Cynthia J. Price, Helen Y. Weng

**Affiliations:** ^1^Department of Biobehavioral Nursing and Health Informatics, University of Washington School of Nursing, Seattle, WA, United States; ^2^Osher Center for Integrative Medicine, Neuroscape Center, Department of Psychiatry and Behavioral Sciences, University of California, San Francisco, CA, United States

**Keywords:** interoception, mental health, emotion regulation, body awareness, embodiment

## Abstract

Emotions are by nature embodied, as the brain has evolved to quickly assess the emotional significance of stimuli and output signals to the body’s viscera and periphery to aid adaptive responses. Emotions involve both implicit bodily and explicit narrative processes, and patients may experience transdiagnostic distress when bodily signals are not attended to and holistically integrated with explicit narratives about experience. Similarly, therapists may be trained in more implicit body-based approaches (i.e., massage/bodywork, physical and occupational therapy, and nursing/medicine) or more explicit narrative-based approaches (i.e., psychotherapy), and may lack training in skills that integrate both levels of emotion processing to aid healing and growth. To address these gaps, we propose a framework where the bridge between implicit bodily sensations and explicit narratives lies in cultivating mindful awareness of bodily sensations associated with emotions. This process brings subjective awareness to notice inner body experience (or interoceptive awareness) that is often outside of conscious awareness, so that it may be understood and re-integrated in more adaptive ways, which we call somatic reappraisal. Using clinical theory and example vignettes, we present mindful interoceptive awareness for adaptive emotion processing as a framework to cultivate and enhance somatic reappraisal. Mindful interoceptive awareness brings more focused and sustained attention to inner body experience; likewise, internal sensations associated with emotions become more granular, vivid, and can shift in ways that facilitate somatic reappraisal. Learning to sustain interoceptive awareness when engaged with mindfulness qualities of nonjudgment and compassion promotes an experience where new associations between emotions, meanings, and memories can be made that generate insights that are holistic and integrative. A clinical vignette is used in this paper to provide examples of this approach in psychotherapy. An example script for use in mindfulness groups is included, and resources are suggested for clinicians to gain more experience. Mindful interoceptive awareness for adaptive emotion processing is a clinical process that can be learned and applied by a range of clinicians to treat mental and physical health conditions that may benefit greater embodied awareness.

## Introduction

Clinical interest is growing in how mindfulness-based interventions may cultivate interoceptive awareness, and may thus aid emotion recognition, processing, and regulation in a variety of mental and physical health conditions (Farb et al., 2015; Khalsa et al., 2017; Weng et al., 2020a). Interoceptive signals are linked to wellbeing appraisals and associated regulatory responses and are thus important indicators of mental health (Khalsa et al., 2017). Likewise, recent reviews of mindfulness-based interventions for psychiatric disorders highlight the promise of mindfulness-based interventions in mental health treatment (Goldberg and Greene, 2018; Wielgosz and Kral., 2019) and the key role of interoception for emotional awareness (Wielgosz and Kral., 2019).

Interoception refers to the process by which the nervous system senses, interprets, and integrates signals originating from within the body, providing a moment-by-moment mapping of the body’s internal landscape across both conscious and unconscious levels (Craig, 2009; Khalsa et al., 2017). Interoceptive *awareness* is the internal sensory information that reaches conscious perception, and thus allows for a subjective sense of the physiological condition of the body, such as heart beat, respiration, satiety, and the autonomic nervous system sensations related to emotions (see definition in Mehling et al., 2012). There are overarching processes involved in interoceptive awareness, such as sensing, interpreting, and integrating sensory information (Vaitl, 1996; Cameron, 2001; Verdejo-Garcia et al., 2012). Related are multiple aspects of interoceptive awareness that reflect the skills involved, such as body literacy which is the ability to verbally identify and describe sensation (e.g., pressure, tingling, and temperature; Price, 2005; Price and Hooven, 2018), and discrimination which is the ability to locate sensations in specific regions of the body (see Khalsa et al., 2017 for a comprehensive list of other aspects). Interoceptive awareness also varies in quality of attention including how focused, sustained, and nonjudgmental it is (Mehling et al., 2012; Price and Hooven, 2018). The act of focusing present-moment attention on internal sensory experience is the basic process of interoceptive awareness and is, as such, an integral component of mindfulness practice (Kabat-Zinn, 1990. Mindfulness-based interventions train qualities of interoceptive awareness that aid focused, sustained, and nonjudgmental attention to regions of the body. This allows more internal sensory information to enter conscious awareness that is sustained over time, while thought-based narrative processes become more muted (Farb et al., 2012). Greater sensory information within conscious perception allows for the possibility of a deeper subjective experience of interoceptive information, involving sensory signals from the viscera and physiological signals associated with emotions, which we call *interoceptive experience*. When mindful attention is brought to interoceptive experience, somatic reappraisal may be engaged by learning to re-interpret internal bodily sensations with less judgement and more acceptance and by experiencing shifts in interoceptive experience.

While many psychotherapeutic approaches utilize mindfulness and attention to interoceptive signals to inform psychotherapeutic process, there is little emphasis on the role of sustained interoceptive attention to a specific region of the body. The ability to sustain nonjudgmental attention on interoceptive experience can facilitate increased emotional awareness and engage somatically based reappraisal processes in therapy that promote wellbeing as described for the therapeutic approach Mindful awareness in body-oriented therapy (MABT; Price and Hooven, 2018). Mindful awareness in body-oriented therapy has been studied in multiple populations with successful implementation feasibility and promising effects (Price, 2005, 2013; Price et al., 2007, 2012, 2020) and is an evidence-based adjunctive treatment for substance use disorder (Price et al., 2019). In this paper, we describe therapeutic skills that develop client interoceptive awareness with the aim of deepening emotional awareness using this process of sustained interoceptive attention. Specifically, we describe how interoceptive awareness aids adaptive emotion processing *via* emotion recognition, processing, and regulation. This process can be integrated into psychotherapeutic practice, and we include clinical vignettes from a psychotherapy session to show how interoceptive awareness and facilitating the capacity to sustain mindful interoceptive awareness can be implemented to promote adaptive emotion processing.

We propose a framework where mindful interoceptive awareness can help de-couple maladaptive sensory-meaning associations (e.g., emotions), and provide the opportunity for new associations to be made. Mindful attention or mindfulness may be understood as *qualities of attention* that are intentional, involve sustained present moment awareness, and include attitudinal qualities of nonjudgment, curiosity, openness, and self-compassion (Kabat-Zinn, 1990; Germer and Neff, 2013). Mindfulness applied to bodily sensory experiences associated with emotional experiences may open up an “experiential space,” where internal sensory experiences and associated meanings become more granular, vivid, and malleable to new interpretation. In this framework, we describe how mindfulness can cultivate insight – which in this context is defined as creating new adaptive interpretations *arising from* and *informed by* bodily sensory information. For clarity, we will refer to this process as *somatic reappraisal*. This is in contrast to interpretations being incongruently *applied to* bodily information (resulting in an emotion-narrative mismatch) or *simply observing* bodily emotional information (which long-term may result in depersonalization or disconnection from the body; Sedeño et al., 2014). In particular, kindness and compassion may facilitate more adaptive interpretations that connect difficult sensations to meaning-making and growth (Gilbert, 2009; Germer and Neff, 2013; Weng et al., 2013; Kirschner et al., 2019), which would improve emotion-narrative congruence (or “integrated sense of self”). As the clinician serves as an embodied model and guide throughout this process, this paper aims to educate clinicians from a variety of backgrounds in: (1) the interplay between emotions and experience of interoceptive sensations, (2) the clinical utility of mindful interoceptive awareness for emotion processing, (3) the importance of sustained interoceptive attention for somatic appraisal, (4) a framework for understanding “experiential space,” and (5) clinical vignette examples of how to facilitate sustained interceptive attention within the context of a therapeutic interaction.

### Understanding How We Feel: Emotions in the Body and Interoceptive Awareness

Emotions arise from appraisals of situations, physiological responses to aid homeostasis, and subjective experiences (Barrett et al., 2007). Emotions have a bodily component, as they arise from neural processing of how the external and internal environments are aligning with internal needs and goals, involving signals sent quickly throughout the body to aid adaptive responses (Damasio, 1999; Phelps and LeDoux, 2005; Smith and Lane, 2015). Emotions are thus by nature *embodied*, as the brain has evolved to quickly assess the emotional significance of stimuli and output signals to the body’s viscera and periphery *via* the autonomic nervous system, including to the heart, lungs, gut, face, and limbs (Phelps and LeDoux, 2005; Sapolsky, 2005; Fox et al., 2018). For example, the centromedial nucleus of the amygdala has direct projections to brain regions, such as the lateral hypothalamus, basal forebrain, and brainstem, which mediate physiological responses associated with emotions *via* activation of the sympathetic nervous system and send signals that impact circulatory, digestive, reproductive, immune, and endocrine systems to aid individuals in allostatic regulation and behaviors (Davis and Whalen, 2001; Phelps and LeDoux, 2005; Seth et al., 2011). Recent research (Kleckner et al., 2017; Salamone et al., 2021) highlights the relationship between interoception and allostasis for effective emotion processing and regulation. Further, emotional events occur with the body situated in space and time, and may include external sensory information, including visual stimuli, social context, and additional information, such as bodily position, tone, touch, and pain. In addition to efferent neurophysiological pathways, interoceptive pathways exist where sensory information from the viscera and periphery are fed back to the brain, mediated in part by the insular cortex (Craig, 2009; Namkung et al., 2017). This pathway allows individuals to engage in interoceptive awareness and consciously attend to signals from the internal environment of the body (Craig, 2009; Farb et al., 2015; Khalsa et al., 2017).

However, sensations in the body are often misinterpreted, avoided, or adequately subtle that people can ignore or be unaware of them. We posit that emotional signals in the body often cannot fully be resolved until an adaptive behavioral output is addressed. For example, a person’s heart will race when running from a car if at risk of being hit, and the person’s heart rate will return to baseline once they have stopped running and feel safe. However, situations are more complex and nuanced, and occur within multi-layered social and cultural contexts, where people may not be able to: (1) fully process the emotional meanings of complex multi-faceted situations and (2) enact agency and control to adaptively act in the moment. For example, an employee may be reprimanded by their boss about a situation they were not aware of and may not have the full understanding and agency to defend themselves in the moment. In this example, the emotions are complex involving anxiety, shame, anger, and disappointment. If an emotional event holds significant relevance to a person’s wellbeing (even if it is outside of consciousness), we posit that without an adaptive internal (e.g., acceptance of the situation and the conscious decision to not address it) or external behavioral expression (e.g., having a conversation with the boss to correct the problem), that emotional representations may remain in the brain and body. For example, unconscious perception of negative stimuli results in “affective coloring” of subsequent neutral stimuli by being rated as more unlikeable (Lapate et al., 2014). This suggests that in the absence of conscious awareness, negative affective information may continue to influence perception.

In the above example, the employee later noticed a lingering sense of abdominal heaviness. Over time and with a repeated similar pattern of avoiding conflict, the client may notice interoceptive sensations (i.e., abdominal heaviness in this example but could be also expressed as muscular tension or some other sensory signal). The client may or may not be aware of these interoceptive signals and may also misinterpret them. Experiential psychotherapeutic processes help clients identify the “felt sense” or the bodily sense of emotional experience (e.g., Focusing; Hendricks, 2001; Gendlin, 1996) and mindful awareness of somatic experience (e.g., Hakomi; Kurtz, 1990). These approaches can facilitate client interoceptive awareness, emotional awareness, and stimulate deepening of psychotherapeutic processes. We propose it is also helpful for clients to learn to mindfully sustain attention to interoceptive sensations in specific bodily regions, and that doing so can further promote awareness and somatic reappraisal.

### Emotions, Thought-Based Narratives, and Maladaptive Associations With Sensory Information

When emotional events happen, sensory experiences are *bound together* with meanings, implicit interpretations, and explicit thoughts and narratives; such sensory-meaning associations may range in how adaptive they are for later functioning, based on both implicit and explicit interpretations that are constructed about the self, others, and the world (Gross, 2001; Gyurak et al., 2011; Resick et al., 2016), reflecting a person’s biological and social history. For example, two people may be in a car accident and break their leg yet have different interpretations of their situation. One may experience fear and pain in the moment, but after adequate medical care and social support, she interprets her situation as fortunate due to the receipt of good care and not being more severely injured. In contrast, the other person had a similar accident but has a history of childhood injury that includes a complex recovery process with dysfunctional family dynamics. He may interpret the situation as much more traumatic and have a more difficult recovery process with re-experiencing of fear, continued pain, and distrust of social support and medical providers. These differing interpretations may in turn lead to different profiles of psychological and physical functioning (Folkman et al., 1986). Complex sensory-meaning associations are often unconscious, habitual, repetitive, and difficult to shift (i.e., rumination; Nolen-Hoeksema, 2000), even when people intellectually “know” they should be “over” something. Clients may think of more positive thoughts and narratives (“I am lucky to be alive, and I will recover in time”), yet cannot internalize these thoughts so that they translate into an associated positive emotional experience, resulting in an emotion-narrative mismatch which can lead to more distress.

Thought-based narratives of emotional experience likely involve the Default Mode Network (DMN), a large-scale brain system that is active when people are engaged in internally directed or self-generated thought (Andrews-Hanna et al., 2014). The anterior insula, a core region in the Salience Network involved in interoception (Craig, 2009), may serve as an integral hub in mediating dynamic interactions between other large-scale brain networks, such as the DMN (Simmons et al., 2013), where its core function is to mark salient events for additional processing and initiate appropriate control signals (Menon and Uddin, 2010). Thought-based narratives may be habitually coupled to certain interoceptive cues, and in some cases may be maladaptive. For example, a brief ache in the stomach may become associated with negative thoughts, such as “I’m so anxious about this that I do not know what to do,” and contribute to anxiety disorder systems. Further, these associations are likely developed and maintained at an unconscious level, as in body memories arising from post-traumatic stress (van der Kolk, 2014).

Negative emotions are not maladaptive in and of themselves; sadness, anger, fear, and disgust all have evolutionary purposes to help people survive, by indicating a problem with the environment and preparing the body for action. However, negative emotions associated with certain events can result in rumination, or the repetition of negative feelings and thoughts, and are a major risk factor of anxiety and depression (Nolen-Hoeksema, 2000). This clinical issue, where maladaptive sensory-meaning associations are strong and resistant to change, is a core feature for many clinical conditions, such as depression, anxiety, traumatic stress, and chronic pain. Rumination is a strong characteristic of psychiatric disorders (Nolen-Hoeksema, 2000) and may reflect paying more attention to the narrative than to sensory information and is often accompanied by well-developed coping patterns of avoidance or bodily dissociation (Price, 2007). We posit that adaptive mechanisms can also exist within persistent negative emotional signals and awareness of sensations from particular bodily regions may enhance emotional awareness. Maladaptive associations between interoceptive information and thought-based narratives may potentially be de-coupled and reappraised using mindful interoceptive awareness. When attention to interoceptive information is sustained, sensory information may be brought into primary focus, while thought-based narratives are dampened, and nonjudgmental attention may aid in creating new affective associations with internal sensory information. This may be an alternate route to reappraisal that is somatically based rather than thought-based cognitive reappraisal, which is mediated by the medial and lateral prefrontal cortex (Ochsner and Gross, 2005; Buhle et al., 2014).

Clinicians from a range of fields and backgrounds (e.g., psychotherapists, body-oriented therapists, doctors, nurses, and physical and occupational therapists) may encounter the clinical phenomena where patients have difficulty engaging in adaptive behaviors because of maladaptive associations made with bodily sensations. Practitioners and therapists trained mostly from a body-based perspective may need to increase their understanding of how bodily sensations are often closely tied to emotions, and to learn verbal and emotional processing skills. Likewise, psychotherapists who are trained in cognitive approaches and talk-therapy may need to increase their understanding of how emotions are often tied to bodily sensations. In both cases, therapists can learn new skills to help their clients bypass word-based cognitions as the initial orientation for self-understanding and develop their capacity to *feel* (their bodies and their emotions). One way to do this is by integrating interoceptive awareness and mindfulness processes to facilitate more sustained and nonjudgmental acceptance of feeling states.

## Emotions and the Body

Emotions result in coordinated responses across the entire body and awareness of emotion can inform adaptive action. We highlight regions in the “core” of the body, including the chest (heart/lungs) and abdomen (gut), involving organ systems that play a “coordinating” role in response to emotion across the body. The lungs indicate respiratory needs and overall integrity within the organism, and respiratory patterns, such as breath rate change depending on emotional states, such as fear, anxiety, and relaxation (Homma and Masaoka, 2008). The heart provides blood circulation to the entire body. Heart rate and heart rate variability are impacted by social emotions and social interactions (Schwerdtfeger and Friedrich-Mai, 2009; Kok and Fredrickson, 2010). Finally, the gut has many bidirectional connections with the brain and is so complex that the enteric nervous system has been identified, a “second brain” system that contains as many neurons as the spinal cord (Mayer, 2011). The gut represents digestive integrity within the human and is sensitive to emotional responses, such as fear, anxiety, and disgust, as digestive processes are decreased when faced with stimuli of threat (Davis and Whalen, 2001; Sapolsky, 2005). The gut is also associated with “gut feelings” that contribute to intuitive decision-making (Damasio, 1999; Mayer, 2011). The microbiome, or bacterial flora within the gut, may also contribute to emotional signals accessible by interoception (Foster and McVey Neufeld, 2013). There is clinical (Lowen, 1967) and research evidence that certain types of emotions tend to be experienced in certain bodily regions (i.e., anxiety in the abdominal region; love and sadness in the heart region; Nummenmaa et al., 2014).

Common clinical examples include the experience of emotions linked to areas of the body where there was an injury (e.g., fear and/or anxiety associated with the neck after whiplash resulting from a car accident) or where the stresses of daily life may influence musculature of the body (e.g., sense of aggravation associated with tightened muscles in the mid-back after a challenging interpersonal interaction with a family member). Facial muscles also indicate emotional states subjectively to the self as well as others (Ekman, 1993). Thus, depending on a patient’s history and context, any area(s) of the body may be implicated as far as the expression and experience of emotional information and thus may benefit from mindful attention. Research experience based on MABT studies (Price, 2005, 2013; Price et al., 2007, 2012, 2019, 2020) suggests significant individual variation. Some of this variation is based on the context of the emotional “injury” – for example, if there was a related physical event (e.g., physical assault, sexual abuse, and injury due to an accident or repeated surgeries) and thus a location on/in the body associated with the emotional experience. It is for these situations that areas in the body outside of the core are particularly implicated (arms, legs, hands, and feet).

## Development and Importance of Sustained Interoceptive Attention

Explicit training is often required to develop the capacity for sustained interoceptive attention, particularly for individuals who avoid or are disconnected from their bodies, to learn *how* to access and attend to inner body experiences (Price and Hooven, 2018). Many experiential psychotherapy approaches involving elements of mindfulness, and mindfulness-based interventions, train people in nonjudgmental awareness of the body, in contrast to avoidance of or reactivity to bodily information that can worsen clinical outcomes. However, many of therapeutic approaches involving elements of mindfulness use a body scan or awareness of the breath as the primary activities to promote sensory or interoceptive awareness, and do not typically teach sustained attention to other regions of the body. For example, people may not learn how to bring mindful attention to areas of the body associated with their own personal stressors and emotions, such as the inner bodily core (e.g., chest and abdomen) or other areas of the body (e.g., areas of tension; in a limb). Although bodily awareness may be cultivated through the body scan exercise, this practice can result in attention passing through areas of the body relatively quickly rather than cultivating the capacity to focus attention in a specific region in the body, exploring and maintaining sustained attention in certain areas where needed. In addition, with the group format of many mindfulness approaches and interventions, teachers/therapists often do not know what the interoceptive ability is of the student/client, how to help them learn and develop the skill of interoceptive awareness or the capacity for sustained interoceptive attention, and how these capacities may increase the potential for better contact with emotional experience.

Thus, to successfully engage in sustained interoceptive attention requires a set of skills. These include the ability to: intentionally move attention into the body (in order to sense), identify sensations, sustain interoceptive awareness, and reflect on and evaluate what is noticed (in order to interpret and integrate; Mehling et al., 2011; Price and Mehling, 2016; Price and Hooven, 2018). The specific skills needed overlap with efforts to develop a taxonomy and measures of interoceptive awareness as they relate to mental health (Khalsa et al., 2017). Importantly, the interpretation of interoceptive signals is necessary for adaptive functioning, and research suggests that dysfunctions in interoception (i.e., disturbance in interoceptive ability, such as interoceptive awareness) may contribute to symptoms of anxiety, mood, eating, addictive, and somatic symptom disorders (for review, see Khalsa et al., 2017). Likewise, adaptively using skills related to interoceptive awareness may reflect psychological wellbeing (Hanley et al., 2017) and be conditioned by mindfulness (Tsur et al., 2016).

**Figure 1:**
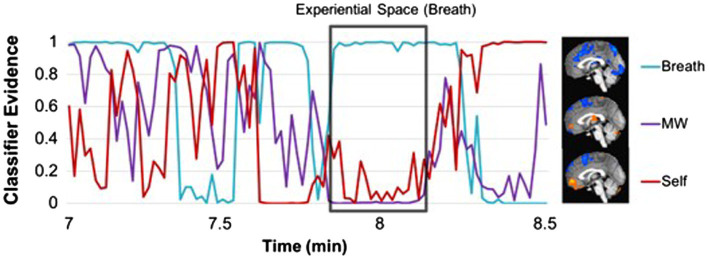
Experiential space and somatic reappraisal: components that contribute to awareness and integration.

Specifically, integrating sustained interoceptive attention into therapeutic approaches involves teaching clients to bring their mindful awareness to a specific region of the body where the client experiences or possibly stores emotional content, and then resting their attention in this region of the body with sustained mindful interoceptive attention. We define the region of focused attention as an “internal experiential space.” This is similar but distinct from the concept of the “felt sense” in Focusing (Gendlin, 1996) that refers to a sense of a meaningful body sensation but often one that is “unclear or elusive, temporary or hard to describe.” The “felt sense” is often the starting place sense that there is somatic information to attend to. The process of bringing attention to an “internal experiential space” refers to more of a deep dive involving sustained mindful attention in a specific region of the body, involving a shift into a meditative state and the ability to maintain mindful attention for an extended period of time (Price and Hooven, 2018). With sustained attention, one can more deeply engage in, i.e., be connected to, their interoceptive experience which allows for more sensory information to come forward and often leads to a positive shift in physical and emotional experience (Price and Hooven, 2018). Mindful qualities of attention, such as curiosity, self-compassion, and a nonjudgmental approach, help individuals access the “raw data” of their sensory experiences, and to attend to and begin to de-couple these from habitual narrative thought. It is thus through the process of sustaining attention in a specific body region/internal experiential space that facilitates the shift from simply noticing sensation to engaging in emotional content or the experience of arising meaningful information (e.g., insight) that underlie somatic reappraisal; see Figure 1.

## Sustained Interoceptive Attention Facilitates Somatic Reappraisal Processes

The processing and integration of experience, particularly responses to situations and interactions that are highly positively or negatively charged, are critical for the somatic reappraisal process. Reappraisal and insight are thought to be key elements underlying certain mindfulness-based approaches that promote a sense of wellbeing and healthy behaviors (Dahl et al., 2015). Cognitive reappraisal is the process of re-interpreting an experience such that the emotional response changes, and is linked to emotion regulation (Ochsner and Gross, 2005). Insight is a sudden shift in perspective or sense of knowing that enhances self-understanding (Kounios and Beeman, 2014). We use the term “somatic reappraisal” to refer to similar processes that arises from interoceptive attention to an internal experiential space. With the focus on client sustained interoceptive attention to *feel* the sensations and/or emotions, any related shifts that may follow are palpable. Likewise, the application of cognitive frameworks of any related insights or associations in meaning allows for healthier intra- and interpersonal responses (see vignette examples below).

## Experiential Space

**Figure 2:**
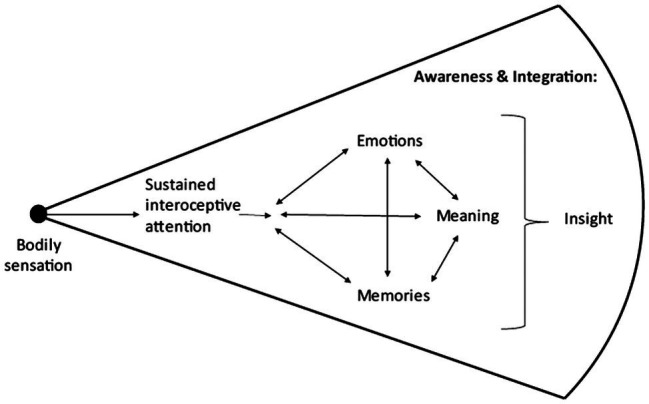
Initial empirical evidence of experiential space during mindful attention to the breath.

Initial evidence for the presence of experiential space, where conscious awareness is characterized by greater internal sensory experience and decreased thought-based narrative, is suggested by neuroscience studies of mindfulness interventions and practice. Mindfulness meditation practice (particularly focused attention to the breath, a form of mindful interoception practice) is associated with greater activation in neural networks associated with interoception (Farb et al., 2013; Fox et al., 2016), and decreased activation of the DMN (Brewer et al., 2011; Fox et al., 2016) which is occurs in mind wandering and self-referential processing (Andrews-Hanna et al., 2014; Christoff et al., 2016). Further, mental states associated with mindful experiential space (greater interoception, decreased mind wandering, and self-referential processing) can be recognized and tracked during meditation using machine learning applied to neural data (Weng et al., 2020b). Using classifier evidence, a continuous measure of the presence of each mental state, initial empirical data show that the presence of experiential space may be possible during a period of breath-focused meditation (Figure 2). For a sustained period of time (about 10min), the classifier evidence for breath attention is very high, while classifier evidence for mind wandering and self-referential processing is relatively low, showing the potential of neural representations of sensory information to become relatively high and sustained, while thought-based narratives may become more muted.

Various medical and psychological approaches, and spiritual traditions, involve attention to “spaces” in the body, including the chakra system developed in India and comprised of seven key electromagnetic centers in the body. The chakras have been also described as Qi – the energy circulating throughout the meridian system in Chinese Medicine (Kaptchuk, 1983). These energy systems vitalize the body and influence our emotional and physical health. Bioenergetics, a body-psychotherapy approach developed by Wilhelm Reich and Alexander Lowen (Lowen, 1967), is also based on the concept of energy or life force and focuses on helping to identify and release blockages in the tissue or body that restrict life force and impede embodiment. In Western medicine, we have physiological evidence of emotions being processed through physical channels (Davis and Whalen, 2001; Phelps and LeDoux, 2005). There is also research demonstrating awareness of emotions felt in the body, as indicated by color maps drawn by participants to indicate different emotions (Nummenmaa et al., 2014) and by identifying emotions and body awareness in a brain mapping study (Terasawa et al., 2013).

## Clinical Vignette Highlighting the Facilitation of Sustained Interoceptive Attention

Our overarching goal for this paper is to present a conceptual framework and clinical vignettes that highlight the critical role of sustained interoceptive attention of emotional information to enhance mental health in therapeutic encounters. Within the realm of psychotherapy, emotions are often addressed using talk-based therapy, and this clinical framework points to additional skills for therapists to learn and incorporate in practice. This approach is in congruence with experiential psychotherapy (e.g., Kurtz, 1990; Gendlin, 1996) that incorporates somatic awareness and mindfulness to facilitate emotional awareness, and emotion-focused therapy approaches, which treat emotions as evolutionarily beneficial for providing valuable information to inform decisions (Greenberg, 2004), and dialectical behavioral (Linehan, 1993) and acceptance and commitment therapy (Hayes et al., 2011), which teach emotion tolerance and regulation skills using mindfulness techniques. Below are short segments from a clinical vignette; please note that this is a composite vignette and does not represent a transcribed clinical interaction.

### Facilitating Interoceptive Awareness in a Specific Body Region

In this example below, the client is a 50-year-old male with low back pain who tends to avoid attending to his sensations (e.g., his back pain and his emotions). He is in his third session with a therapist. The therapist facilitates client interoceptive awareness in his back where he has symptoms of pain, to help him to “hear” and to feel (*vs*. avoid) his internal experience/emotions.

**Therapist:** I’d like to invite you to bring your awareness into your low back. To do this, feel where your low back is in contact with the chair. Can you feel that?

**Client:** Yes.

**Therapist:** Great, now allow your inner attention to move down toward this region of your body. To do this, let your attention gently move down through you until you feel like you’re inside this region of your low back, from the inside. Take your time so that you move your attention slowly, allowing yourself to track where you are on the inside so that you know when you have arrived in this internal space.

There is a pause as the client tunes attention inward and they both track his internal presence.

**Therapist:** Let me know when you feel like you’ve arrived inside the space of your low back.

**Client:** I’m having a little trouble. I think I’m feeling nervous.

**Therapist:** You’re noticing that you’re nervous…

**Client:** Yea, maybe about bring my attention close in to my back.

**Therapist:** Um hm. Can you gently place a hand on your lower abdomen? This will help you guide your attention into this general area of your body. Does this feel okay?

**Client:** Yes, that’s fine.

**Therapist:** Okay. Do you feel the sensation of nervousness inside in this area?

**Client:** Yea. It’s like a barrier goes up.

**Therapist:** Can you feel the sensation of a barrier inside?

**Client:** Yea, it’s like a block right in my middle – just above where my hand is.

**Therapist:** Great noticing. Can you move your hand up a bit to that middle place?

**Client:** Um hm.

**Therapist:** How is that?

**Client:** That’s okay.

**Therapist:** Can you just bring your attention right into this region and be with this feeling of an interior barrier?

{Client nods}

**Therapist:** Just let your attention sink into this place in your body and with this feeling of the barrier.

{Long pause as client does so; therapist assesses that client presence is inside}

**Therapist:** What are you noticing now?

**Client:** The barrier is like a dark band. I can feel the pain of my back right there too.

**Therapist:** You are noticing a dark band and the feeling of pain. Can you tell me more about the sensation of the pain? What does it feel like?

**Client:** It feels like pressure and ache.

**Therapist:** Good noticing. You are doing a great job staying with this. What else do you notice here?

**Client:** The internal pressure feels like a heavy brick inside. I guess that’s part of the discomfort too.

**Therapist:** Can you bring your attention toward the heavy brick feeling? Just let yourself feel the heaviness. Allow yourself to feel how it is inside this place…

{Client sighs deeply and lips tremble}

**Therapist:** What are you noticing now?

**Client:** I feel so overwhelmed with stress, like it’s going to swallow me up…

**Therapist:** I’m noticing your face while you say this – this sounds really hard…

**Client:** Yea, I guess I’m feeling how hard this is. How worn down I feel - and it’s kind of emotional.

**Therapist**: That is great noticing ~ let yourself continue to feel the emotional quality of this…

### Facilitating Sustained Interoceptive Attention in a Specific Bodily Region

The therapist continues by coaching Client 1 in sustaining interoceptive attention. Doing so facilitates the client’s experience of new sensory information, leading to an emotional release that takes the client out of a state of mindful attention or presence. The therapist notices this and gently facilitates the client’s return to interoceptive awareness and a maintained focus on sensation. The client experiences both positive emotional and physical shifts.

**Therapist:** You are doing a great job feeling the heaviness – and this experience of overwhelming stress that you feel in your body. Are you okay continuing to focus in this place for a few more minutes?

**Client:** Yes, it’s okay.

**Therapist:** Alright, just continue to maintain your presence inside where you feel the sensation of heaviness. Let this be as if you are keeping yourself company inside – keeping this heavy place company.

{There are a couple minutes of silence in which the client is attending to internal experience. Some tears fall down the client’s cheek…}.

**Therapist:** You’re doing a great job just allowing yourself to feel….

{Client’s attention flits out of inner attention, and his eyes open briefly.}

**Therapist:** See if you can let you attention come back down into this area of your body. Just continue to stay inside in a gentle way with yourself.

{Client is able to re-engage with interoceptive attention to inner area of body}

**Therapist:** Great job. Now, again, just bring your attention into this area and check in again with what you notice.

**Client:** The heaviness is still there but it feels smaller.

**Therapist:** It feels smaller. Okay, can you describe that?

**Client:** It doesn’t take up as much space – like it’s just a more dense ball instead of the full area.

**Therapist:** It’s smaller now. Anything else that you notice?

**Client:** I feel more calm inside. Like I released some of the constant worry.

**Therapist:** That’s wonderful that you feel calm. Can you also bring your attention to where my hand is on your back and let me know how your back is feeling?

**Client:** Huh…. You know when we started it was really achey feeling there and now I don’t feel that achiness any more.

**Therapist:** You are noticing that the achiness in your back has changed.

**Client:** Yea – and I feel like I can breathe more fully too.

**Therapist:** That is great noticing. Why don’t you take deep breath and notice how it feels in your low back when you do that.

**Client:** {takes a deep breath and smiles}: *Wow, haven’t done that in a while ~ it feels good to breathe so deeply…*.

## Embodied Emotional Responses Are Unique To Each Individual and Context

Social norms regarding the processing of emotions based on culture, family history, and social roles can impact interpretation of emotional events and emotional expression (Mesquita, 1996). In addition, many people have never been explicitly taught or given models of adaptive emotional processing. Also, there are individual differences based on biological sensitivity and personality traits, and thus, there is a wide spectrum in interoceptive awareness (e.g., being sensitive to, able to access, interpret, and adaptively respond to bodily information; Farb et al., 2015).

### Clinical Vignette

The client from vignette above grew up with an alcoholic father where emotions were not processed in an open and healthy manner, contributing to his sensitivities, regulatory skills, and family dynamics. The therapist asks for clarity and more information about the client’s concerns about his drinking behavior, and by so doing gains a better understanding of the family history and current events/interactions that may be impacting the client’s back pain.

**Client:** I guess I’m a bit concerned about drinking too much and my wife recently mentioned that she was too…I have alcoholism in my family so I’m kind of sensitive to this.

**Therapist:** What do you mean by “sensitive?”

**Client:** Oh, I mean that I don’t want to drink too much; to be like my brother or my dad.

**Therapist:** Ah, thanks for the clarification and yes, that is important. Is there anything else going on with your relationships with your family right now?

**Client:** Yea, actually. My parents are quite elderly and they are needing increasing care. My brother and sister and I are having more contact trying to sort out what to do, and who’s going to do what. We are not agreeing about much. The other day I got so mad at my brother that I hung up on him. It’s not pretty.

**Therapist:** I’m sorry, that sounds very stressful.

**Client:** It is. Not helping my back any, either.

## Embodiment, Sense of Self, and Somatic Reappraisal Processes

Embodiment theory relegates consciousness to the body as an “integrated function” (Hendricks, 2001; Gendlin, 1996), in line with theories of Merleau-Ponty who described the importance of the unity of body and mind and our bodies as the perceived medium whereby our world comes into being (Merleau-Ponty, 1971). Likewise, the theoretical basis of experiential psychotherapy approaches is that healthy functioning results when as many aspects of the self as possible are integrated in awareness; the term “self” refers to the implicit organization of aspects of the self that serves to integrate experience and create a sense of self, an ongoing and constructive process (Greenberg and Van Balen, 1998). Embodiment is thus a process of becoming that is fundamentally linked to interoceptive awareness (Herbert and Pollatos, 2012) – providing a “revised viewpoint on concepts of the person” (Strathern, 1999), p. 198). Connecting with internal experiential spaces in the body is a process by which we can experience the link between physical sensations and emotions, but it is through sustained interoceptive attention in specific regions of the body that shifts occur in physical and emotional sensations. These shifts in interoceptive experience are often imbued with meaning and lead to insight and involve somatic reappraisal (Price and Hooven, 2018; see Figure 1). The focus on sustained mindful interoceptive awareness in a specific internal experiential space is therefore designed to facilitate client interface with immediately sensed, but implicit, experience to enhance awareness of self and, thus, wellbeing.

### Clinical Vignette

In the continued clinical vignette below, the therapist provides a simple conceptual framework for the therapeutic process and facilitates a review and client reflection on his experience of sustained mindful interoceptive attention. The process of client reflection on interoceptive experience is critically important for facilitating evaluation and integration of experience that is commensurate with somatic reappraisal processes.

**Therapist:** This practice, of attending to your inner experience can bring more awareness to how you feel and also facilitate shifts, like you had today. What was new or surprising to you in this experience?

**Client:** Well, I am surprised at how good I feel right now. But mostly I realized how much my stress is the primary issue and that it’s likely connected to my low back pain. I hadn’t realized that before – all the stress I feel with my parents – I hadn’t thought about it affecting how I feel in my body. I’ve had back pain in the past so I figured this was just a reoccurrence of that. Maybe I’d pulled something in my workshop, I don’t know….

**Therapist:** Yea, and it could be both but made worse by the stress. But it sounds like there is a connection there, and this is greater awareness of this for you. You got in touch with some emotion and that sense of heaviness and feeling overwhelmed.

**Client:** Yea, that was something! I’m not used to showing my emotions so I hope that was okay?

**Therapist:** That was very okay, I’m glad you felt safe enough to do so. Knowing the depth of your feelings can be very helpful as you integrate this information and let it guide you.

**Client:** Yea –I need to be more careful about how much of this stress I take on in my family. I want to start paying more attention to my body too - so that I can better notice the connections with my stress levels. And also I love this light feeling and I don’t want it to go away….I feel so good…

**Therapist:** These are all really important things – finding ways to support yourself in relationship to your family and to know how “good” can feel. We can talk about some ways you can help yourself pay more attention to your body, and also your emotions, in your daily life.

**Client:** Yea, I’d like that.

## Practicing Interoceptive Awareness in Daily Life

The integration of interoceptive awareness into daily life is critical for increasing attention to and awareness of somatic information about how we are feeling and responding to our environment and relationships. Doing so helps to develop and set healthier habits and a more resilient regulatory response to stressors. It may take some time to learn to access and sustain mindful interoceptive awareness, so clients can be encouraged to practice on their own. Daily practice enhances interoceptive awareness skills and may also encourage bringing mindful attention to everyday actions. The role and importance of interoceptive awareness practice is apparent in research that highlights maintained positive longitudinal outcomes among samples that report consistently high continued practice of interoceptive skills (Price et al., 2012, 2019). In addition, qualitative studies indicate the importance of interoceptive awareness practice for increased self-agency, embodiment, and skills for processing and coping with challenging emotions (Price et al., 2011; Price and Smith-DiJulio, 2017).

## Summary, Discussion, and Further Recommendations

This paper outlined the clinical rationale and vignettes of applying mindful interoceptive attention to facilitate adaptive emotion processing. Attending to sensations through sustained attention in an internal experiential space supports the malleability of connections between and interpretations of experiences, so that adaptive somatically informed reappraisal may occur. Finally, these adaptive reinterpretations of experiences that are rooted in bodily experience support a fuller sense of embodiment and a more integrated sense of self. Importantly, we consider adaptive emotion processing using mindful interoceptive awareness as a transdiagnostic clinical tool that can be applied to a range of psychiatric and physical health conditions that can benefit from mind-body interventions. Because many psychiatric conditions involve maladaptive emotion processing (Davidson et al., 2000; Kring and Sloan, 2009; Gross and Jazaieri, 2014; Khalsa et al., 2017), and physical health conditions may have co-occurring emotional impacts or disorders (such as comorbid depression with cancer), facilitating sustained interoceptive attention may be a useful therapeutic process for clinicians from a range of fields to treat patients (e.g., in psychotherapy, body-oriented therapy, yoga therapy, medical visits, physical and occupational therapy visits, and within mindfulness-based interventions and approaches).

We recommend that clinicians interested in interoceptive awareness should engage in somatic mindfulness practice themselves, which will support clinical skills needed to guide patients as they learn and develop the ability to do this themselves. To name a few resources (which are not exhaustive), clinicians may take courses in group mindfulness-based approaches such as Mindfulness-Based Stress Reduction and Mindfulness-Based Cognitive Therapy (see listings at the Mindfulness-Based Professional Training Institute) at the University of CA San Diego^1^) or in individual somatic approaches that are founded on mindfulness principles, such as Hakomi and Mindful Awareness in Body-Oriented Therapy (MABT; see listings at the United States Association of Body Psychotherapy^2^). As mentioned previously, group mindfulness courses may not give extended mindfulness instructions to other regions of the body (aside from the breath), so we included an example group script with mindful attention to more body areas in Appendix A.

Therapists doing this work will notice that once the client in engaged in sustained interoceptive attention, the client is in a qualitatively different state compared to when the client is in a more “usual” narrative mode or “default mode” (Andrews-Hanna et al., 2014). There are distinct neural patterns associated with different mental states; the mindful interoceptive experiential focus activates regions associated with bodily awareness and executive attention (insula, secondary somatosensory cortex, inferior parietal lobule, and lateral prefrontal cortex), whereas the narrative mode, while thinking about the self, activates hubs of the Default Mode Network, including the medial prefrontal cortex (Farb et al., 2007). One mechanism through which mindful interoceptive awareness may change interpretations is through de-coupling the habitual narratives from present-centered sensory experience so that sensations become the predominant stimuli in conscious awareness (while quieting narrative mode). By bringing other elements of mindful attention, such as curiosity, nonjudgment, and kindness to these sensations (*via* non-verbal modes), new associations may arise so that when clients return to their narrative mode, the thoughts and interpretations are imbued with a sense of acceptance. In this way, the narrative and experiential mode may become more integrated and work together (as opposed to narratives that are at odds with or incongruent with the experiential mode). As exemplified in the clinical vignette, clients are able to re-integrate significant sensory experiences and memories into their life narratives through a lens of self-understanding and compassion. This allows difficult experiences to be held simultaneously with their sense of self (rather in a contradictory push/pull manner), which represents a dialectical approach to holding experiences (Linehan, 1993).

Research is beginning to examine related therapeutic processes and questions. One measurement challenge is that interoceptive awareness is difficult to measure in the moment, particularly the fluctuation of various mental states, such as attending to the body, mind wandering, or engaging in self-referential processing. Mixed-methods research is needed to examine the phenomenology of first person experience and related physiological changes associated with interoceptive awareness in a therapeutic context. Initial clinical studies provide some guidance for future research in this realm. For example, results from a phenomenological study of interoceptive awareness processes highlight how the development of interoceptive awareness interfaces with increased self-referential processing of emotions and behaviors in daily practice between sessions (Price et al., 2011). A more recent study, designed to explore engagement in sustained interoceptive attention during MABT sessions (through therapist or second person report), found a positive relationship between self-referential processing and sustained interoception (Price, 2016). In addition, longitudinal findings from MABT intervention studies highlight the critical role of practice and integration of interoceptive awareness into daily life for maintained health outcomes (Price et al., 2012, 2019), including increased respiratory sinus arrhythmia (a psychophysiological indicator of emotion regulation capacity; Price et al., 2019).

The physiological mechanisms underlying interoceptive awareness are being examined through brain imaging studies. New neuroscientific measures are able to apply machine learning approaches to functional magnetic resonance imaging data to (1) learn participant-specific brain patterns for various mental states (attention to breath, mind wandering, and self-referential processing) and (2) use these neural patterns to decode the fluctuations in internal attention, and estimate amount of interoceptive focus during a 10-min meditation session (Weng et al., 2018). In the future, this approach may be extended to estimate other aspects of mindful awareness, such as being present-centered and compassionate, so that these mental states can be measured with meditation practice and associated with psychological and physical health outcomes. With better theoretical, phenomenological, and quantitative frameworks to characterize mindful interoceptive awareness for adaptive emotional processing, we aim to give clinicians and researchers more tools to help patients improve mind-body health and wellbeing.

## Author Contributions

CP and HW jointly conceived and wrote this manuscript. All authors contributed to the article and approved the submitted version.

## Funding

This manuscript was partially supported by the National Center for Complementary and Integrative Health (NCCIH) at the National Institutes of Health by grants: NCCIH K08-AT009385 (HW), NCCIH T32 AT006956 (F.M.H. and S. Adler). Its contents are solely the responsibility of the authors and do not necessarily represent the official views of NCCIH.

## Conflict of Interest

CP serves as the director of the Center for Mindful Body Awareness, a 501c3 organization that provides educational trainings in the mindful awareness in body-oriented therapy (MABT) approach to therapists/practitioners in the community, at academic institutions, and in clinical settings. The Center also develops, implements, and evaluates programs involving the practice of interoceptive awareness practice in collaboration with community or academic clinical settings. There are no other disclosures reported.

The remaining author declares that the research was conducted in the absence of any commercial or financial relationships that could be construed as a potential conflict of interest.

## Publisher’s Note

All claims expressed in this article are solely those of the authors and do not necessarily represent those of their affiliated organizations, or those of the publisher, the editors and the reviewers. Any product that may be evaluated in this article, or claim that may be made by its manufacturer, is not guaranteed or endorsed by the publisher.

## Footnotes

^1^ https://mbpti.org/

^2^ https://usabp.org/

## Appendix A

**Group exercise script**

**  Mindfulness body awareness for adaptive emotion processing**

**  Description**

This 30–45min exercise may be used in conjunction with and adapted for mindfulness-based group exercises. It emphasizes the connection between sensations felt in the body as they are related to emotional responses, and teaches participants to connect with their bodies to access, recognize, and understand emotional information. An invitation should be made to understand and listen to these signals to inform decision-making.

**  Introducing the exercise**

Emotions help let us know how we are in relation to our circumstances and environment. For example, we may feel sad in response to a loss, and joyful in response to a success. Often, we can feel a mix of emotions. In addition, people differ in how they may emotionally respond, even to similar situations. Emotions have a bodily component, where they can change our heart rate, sweat response, breathing rate, facial expressions, and digestion system. This helps signal to both ourselves and others the state of how we are, and helps prepare our bodies for adaptive movement. Sometimes we are not in tune with our emotions, which may worsen situations or our mental and physical health. To access this information that is meant to help guide, we will practice becoming more aware of our emotions, particularly how they are felt in our bodies, a form of “listening to our bodies.” With continued practice, this may help us access our internal knowledge to make wiser and more holistic decisions in our lives.

**  Exercise**

Let us gently prepare our minds and bodies for this mindfulness exercise. Sit in a comfortable but alert posture. Gently close your eyes. Take a few deep breaths, allowing yourself to let go of any tensions and thoughts you may have with each out-breath. You may relax your face and any other part of your body that feels tense. Set the intention that this time will be dedicated to re-connecting with your own experience as it arises, moment by moment. This is an act of kindness towards yourself in and of itself. Try to bring this feeling of kindness and acceptance to your awareness in each moment.

Settle into your breath…Notice the sensations that accompany your breath…Notice where you feel your breath most clearly…This may be at the nostrils, the throat, the chest, or the belly…Let your gentle awareness sit with your breath where you feel it most clearly…Notice how it feels on the in-breath…and out-breath…

  (pause)

We can learn to be mindful of any object…We have mostly practiced being mindful of the breath and mindful of body sensations…Today, we will practice being mindful of our feelings and emotions, which are tied to our bodily sensations. Sometimes we may feel overwhelmed by our emotions or not be very in tune with them. Mindfulness may help us to become better in touch with our emotions without viewing them as absolute truth or pushing them away. In this way, we may develop a better relationship to our emotional experience

  (pause)

**  [Reflecting on situations, inviting emotions in]**

We all go through different experiences in our lives and have unique responses based on our histories and personal understandings…Bring to mind something that has been going on for you, whether pleasant, unpleasant, or both…Perhaps a transition of some kind, a decision you are trying to make, a relationship issue with a family member, friend, or coworkers…Notice what is coming up for you without needing to solve or change anything…

**  [Face/head/shoulders]**

Emotions manifest in our system as sensations in the visceral body. For example, when we are afraid, our heart may begin to race, our breath may start to quicken, we may begin to sweat, our chests may constrict, and we may have a scared look on our face. So let us tune into the sensations of our body more closely tied to emotions. Let us start with the areas around our face, neck, and shoulders.

Bring your attention to your face…. Our faces signal to ourselves and others how we may be feeling…Notice sensations around your forehead…any tension, tingling, pressure, warmth or coolness…Now notice sensations in your cheeks and jaw…Notice any tension, holding those areas in a certain way…Just gently notice any sensations that arise, with a sense of openness and acceptance…No need to judge or push away the feelings, just notice them…Notice the sensations around your lips and chin…. Now bring your kind attention lower to the throat area…and to the shoulders and back…We can tend to hold tension in our shoulders and back…Just notice any sensations – pressure, tingling…If your mind wanders to something else – thoughts, worries, memories – just gently notice that and bring it back to the sensations of your shoulders and back…

**  [Heart]**

Now let us start with the heart area. Heart sensations can indicate our stress levels as well as how connected we feel to ourselves and others. What kind of sensations do you feel around your heart? Try to notice the sensations without labeling a particular emotion. Do you feel pressure? Tingling? Constriction? Openness? Calm? Warmth? Notice how these internal sensations may change moment to moment. No need to judge or analyze what you are feeling, just observe in a kind way…

  (pause)

**  [Cultivate compassionate nonjudgmental relationship]**

You may feel not much emotion right now at all. This is perfectly fine. Just tune into your heart area and examine whatever sensations arise there…Whatever sensations arise, try your best to approach them with an air of curiosity and openness…

You may notice that your thoughts distract you from your internal emotional experience. When this happens, just gently bring your attention back to the sensations of your emotions with an air of kindness

  (pause)

You may notice that your emotional sensations may feel unpleasant or pleasant. No matter what valence they are, just notice how they change, moment-by-moment. If a feeling ever becomes too overwhelming, you may always turn your attention back to your breath. But if it is not, see if you can approach it with an air of curiosity and openness.

See how your internal sensations may change moment-by-moment…Check in with your heart area now. Do you still feel pressure? Tingling? Constriction? Openness? Calm? If you feel not much at all, this is fine. Just keep your attention in this area and notice any sensations that may arise

  (pause)

Even if you do not like the feelings you are experiencing, see if you can accept them as part of your current experience. This does not mean you are giving into them; it just means that this is part of your experience right now. If you sense yourself resisting, see if you can loosen your resistance. Just examine your visceral sensations, moment-by-moment…

**  [Gut]**

Now let us turn our attention to our gut area…The gut processes lots of information regarding digestion, worry, anxiety, as well as creativity and intuition…Notice any sensations you feel in your gut and stomach area…you may feel pressure, tension, tingling, aches, warmth, or not much at all…Just follow the sensations moment-by-moment in a kind way…See if you can stay with the sensations…

**  [Cultivating equanimous responses to bodily emotional sensations]**

Notice if the sensations are pleasant, neutral, or unpleasant, or maybe a mix…See if you want to cling to pleasant sensations and push away unpleasant sensations…See if you can be open and accepting of whatever feelings you experience moment-by-moment…Even if they are pleasant or unpleasant

  (pause)

**  [Labeling feelings]**

Sometimes we do not understand how we are feeling. Describing feelings with words may help us understand what we are going through…Bring your attention to your body in a more holistic way…perhaps starting at the stomach and gently bringing your awareness to the rest of your body…See if any emotions may be living in your body as I list some possible emotion states…See what words may resonate with you in this moment…

  [Pause between each word – use other words as needed].

“Calm…pleasant…happy…interested…neutral…bored…tension…worry…fear…anxious…sad…” If none of those words resonated, give yourself some time to see if another word fits better…You may not need words at all…Pictures, images, sounds may work better…

**  [Listening to messages from the body]**

As you are present with the sensations and feelings in your body, see if there are any messages your body is trying to tell you…Emotions tell us about our needs in our life…See if any wishes, needs, or boundaries may be coming up…No need to rush…Just ask this of your body, and answers may emerge now or at a later time…See if you can listen in this different way, and notice what may come up… (long pause)

And as we bring this exercise to a close, take a few moments to appreciate the sensations and feelings in your body and how they help guide you…When you are ready, you may open your eyes and attend to your surroundings.
